# Adapting and implementing health systems guidelines: learning lessons from countries

**DOI:** 10.1186/s12961-026-01460-x

**Published:** 2026-09-10

**Authors:** Seraphine Zeitouny, Christine Fahim, Abiodun Olugbenga Adewuya, Samuel Adjorlolo, Chanda Mwamba, Claudia Marcela Velez, Sharon E. Straus, Robert Marten

**Affiliations:** 1 School of Population and Public Health, University of British Columbia, Vancouver, Canada; 2 London School of Economics and Political Science, London, United Kingdom; 3 Knowledge Translation Program, St. Michael’s Hospital-Unity Health Toronto, Toronto, Canada; 4 Lagos State University College of Medicine, Lagos, Nigeria; 5 Department of Mental Health Nursing, School of Nursing and Midwifery, University of Ghana, Accra, Ghana; 6 Centre for Infectious Disease Research in Zambia, Lusaka, Zambia; 7 Faculty of Medicine, Universidad de Antioquia, Medellín, Colombia; 8 Alliance for Health Policy and Systems Research, World Health Organization, Geneva, Switzerland

**Keywords:** Health system guideline adaptation, Health systems guideline implementation, The Alliance for Health Policy and Systems Research, Policymaking, Health systems strengthening

## Introduction

Adapting and implementing evidence-based health system guidelines presents numerous challenges, particularly when they are introduced in settings that differ from those in which they were originally developed [1]. Effective implementation requires careful consideration of local context and the development of processes that enable these guidelines to be adapted to the specific settings and populations [2] Health guidelines traditionally have focused on clinical practice and healthcare service delivery, but there is a growing global recognition of the importance of developing guidelines that address structural and policy-level challenges to strengthen the broader health system [3]. Their integration into policy and practice has been associated with significant improvements in health outcomes for the target populations [4]. Furthermore, effective implementation is critical for strengthening health systems and advancing progress toward the Sustainable Development Goals (SDGs). Despite this, empirical knowledge about the diverse factors influencing the adaptation and implementation of health system recommendations remains limited, particularly in low- and middle-income countries (LMICs) [5].

To address these gaps, the Alliance for Health Policy and Systems Research (The Alliance), with support from the Knowledge Translation Program, the South African Cochrane Centre and the Ethiopian Knowledge Translation Directorate developed a programme of work entitled “Research to Enhance the Adaptation and Implementation of Health Systems Guidelines (RAISE)”. Working together with policymakers and health systems managers, the RAISE initiative explored how health systems guidelines are understood, adapted and integrated into policy and practice. Following an open call for proposals, the RAISE project supported selected research teams in adapting and implementing health systems guidelines across various guideline focus areas in LMICs, including Colombia, Ghana, India, Mozambique, Nigeria and Zambia.

This special issue of Health Research Policy and Systems presents the findings of the RAISE initiative, highlighting a myriad of real-world practices and insights. It documents diverse approaches to support the adaptation and implementation of complex health systems recommendations as well as the integration of guidelines in health policymaking and health systems strengthening efforts. We summarize the findings across countries and consider future research directions in this editorial.

## Findings

Velez et al. conducted a comprehensive analysis of Colombia’s implementation of clinical practice guidelines and 13 of World Health Organization health systems guidelines focusing on governance, financial and delivery arrangements [6] Through document analysis, this study identified access to care as the primary obstacle hindering the effective implementation of guidelines in Colombia. Despite recommendations advocating for approved and publicly funded technologies and services, authors found these essential healthcare resources continued to be inaccessible or unavailable to various segments of the population, mainly those living in rural areas or low-income settings*.* In a successive study conducted by Velez et al. team, the authors engaged with the Colombian Ministry of Health to prioritize two groups of health system recommendations for implementation. Following dialogues with policymakers, the team developed evidence briefs addressing early fibrinolysis for patients with ST-elevation myocardial infarction (STEMI) and rehabilitation services for patients with amputations. The authors used the SUPPORT methodology’s systematic process of searching and synthesizing evidence [7] to inform the planning process. These briefs highlighted the need to improve timely access to rehabilitation services for patients with amputations, particularly for individuals residing in rural areas and low-income settings. Barriers to implementation included patient sociodemographic characteristics and the scarcity of trained clinicians. Findings suggested that establishing integrated healthcare networks between community facilities and hospitals could be a promising strategy for improving health outcomes and patient experiences [8]*.*

Adjorlolo et al. investigated home visit practices in Accra, Ghana, as an integral part of efforts to improve primary healthcare. They explored the challenges encountered by nurses and health professionals during home visits in a rural–urban setting in the Greater Accra region and identified potential solutions to overcome barriers. Their findings revealed challenges related to transportation, access, lack of basic logistics (i.e., home visit bags and essential medical tools), finance and sociocultural practices. For example, poor road infrastructure, the location of communities and a fragmented residential address system—common barriers across many LMICs—made it challenging for staff to locate and conduct home visits. In piloting and developing future home visit guidelines, authors proposed multifaceted measures that could streamline and enhance home visit practices, such as training, the allocation of human and financial resources and the provision of safety and security to male and female staff [9].

Adewuya et al. focused on adapting and piloting the implementation of the World Health Organization (WHO) Guideline recommendations on digital interventions for health system strengthening [10] for mental health in Lagos state, Nigeria. They explored the perspectives and experiences of end-users and delved into the challenge of balancing the universal applicability of guidelines with the need for local adaptation. The guidelines were contextualized and adapted to the specific needs of mental health services. This study proceeded with a pilot implementation across five randomly selected primary care centres to assess the perceived effectiveness, acceptability, appropriateness and feasibility among clients, health workers, mental health professionals and health managers. In addition, health workers’ and managers’ readiness for implementation change was assessed. The authors found that recommendations, such as drug stock notifications, health workers’ supervision and targeted client communication to strengthen digital mental health services, appeared to be effective and feasible. However, health workers expressed reservations about client-to-health provider communication and clinical decision support, highlighting various degrees of stakeholders’ readiness to accept changes to health system guidelines [11].

Mwamba et al. evaluated the adaptation and implementation of Zambia’s multi-sectoral cholera elimination plan (MCEP) using the knowledge-to-action framework [12]. Following a comprehensive review of the MCEP and engagement with key stakeholders, analysis showed that the appointment of a coordinator and the establishment of technical working groups enhanced coordination and supported the case for increased investments. However, slow institutionalization, weak coordination, inadequate funding and poor infrastructure fragmented implementation. The authors suggested that the commitment of all stakeholders, political will, and the allocation of funding to support these plans could help eliminate cholera. They recommended developing a collaborative, multi-sectoral strategy rooted in national guidelines and policies to empower and support communities in eradicating cholera [13]*.*

Finally, Fahim et al. described the implementation and evaluation of a technical support program aimed at strengthening RAISE research teams’ capacity to conduct health system guideline adaptation and implementation research. The support program used an integrated knowledge translation approach emphasizing a user-driven model to determine the priorities, needs and support required from study teams. This model featured an in-person capacity-strengthening workshop, personalized coaching sessions with methods experts and a virtual ecosystem of webinars, discussion boards and workshops. The research teams perceived the program to be of high quality. They identified significant barriers impeding the adaptation and implementation of health systems guidelines, such as conflicting health system and stakeholder interests, the scarcity of available data and equipment for effective data collection and limited capacity and poor knowledge. They also revealed enablers, such as forging partnerships with health system stakeholders at the project’s inception, employing a multi-sectoral approach to implementation and investing in the competencies and skills of their implementation teams [14].

The studies featured in this special issue, funded through the RAISE programme, contributed valuable insights into innovative strategies and approaches for addressing challenges in adapting and implementing health system guidelines in resource-constrained settings. A key element of this programme of work was the engagement with policymakers actively involved at project initiation and throughout the research process. Regardless of the specific focus area of the guideline, the findings collectively emphasized the importance of effective implementation and, crucially, the need for contextualization interventions through early and meaningful stakeholder engagement. These studies highlight the need to address significant barriers to guideline adaptation and implementation, such as limited resources and resource allocation, weak coordination and inadequate infrastructure. They also identify key facilitators, such as the use of evidence briefs, the appraisal of international guidelines to ensure their local adaptability and the use of a user-driven model to build capacity. Multi-sectoral governance and collaboration also emerged as essential components for the successful adaptation and implementation of guidelines across diverse contexts. Many research teams called for ongoing efforts to strengthen research capacity and to synthesize insights from various user perspectives.

In conclusion, while health system guidelines are essential tools for strengthening health systems, their effectiveness depends on context-specific integration into policies and practices. Adapting and implementing evidence-based guidelines requires not only specialized expertise to inform and shape their development but also active engagement with policymakers to ensure they are relevant and actionable. Collaborative, multidisciplinary approaches that consider local needs and capacities are crucial to maximize the applicability, relevance and impact of these guidelines.

## Future research directions

The articles in this special issue demonstrate the value of generating contextualized and policy-relevant research to advance the adaptation and implementation of health systems guidelines. The analyses across countries show varying degrees of stakeholder readiness to embrace changes as well as barriers and facilitators of health systems guideline implementation. This special issue also highlights how common challenges, such as competing health system priorities and stakeholder interest, limited research capacity and the know-how to adapt and implement guidelines. To advance this type of work, there is a need to move towards building early and sustainable partnerships using co-creation and co-production approaches with policymakers, strengthening health systems implementation capacity and supporting guideline implementation in a multisectoral fashion. The papers presented in this special issue also identified an increasingly recognized need to focus on supporting capacity strengthening for health policy and systems research.

## Data Availability

Not applicable.

## Abbreviations

LMICs: Low- and middle-income countries
MCEP: Multi-sectoral cholera elimination plan
RAISE: Research to Enhance the Adaptation and Implementation of Health Systems Guidelines
SDGs: Sustainable development goals
STEMI: ST-elevation myocardial infarction
The Alliance: The Alliance for Health Policy and Systems Research
WHO: World Health Organization

## References

[1] Wang Z, Norris SL, Bero L. The advantages and limitations of guideline adaptation frameworks. Implement Sci. 2018;13(1):1–13. 10.1186/s13012-018-0763-4. 29843737 PMC5975671

[2] Wang Z, Norris SL, Bero L. Implementation plans included in World Health Organisation guidelines. Implement Sci. 2016;11(1):1–9. 10.1186/s13012-016-0440-4. 27207104 PMC4875699

[3] Bosch-Capblanch X, Lavis JN, Lewin S, Atun R, Røttingen JA, Dröschel D, et al. Guidance for evidence-informed policies about health systems: rationale for and challenges of guidance development. PLoS Med. 2012. 10.1371/journal.pmed.1001185. 22412356 PMC3295823

[4] Correa VC, Lugo-Agudelo LH, Aguirre-Acevedo DC, Contreras JAP, Borrero AMP, Patiño-Lugo DF, et al. Individual, health system, and contextual barriers and facilitators for the implementation of clinical practice guidelines: a systematic metareview. Health Res Policy Syst. 2020. 10.1186/s12961-020-00588-8. 32600417 PMC7322919

[5] Breneol S, Curran JA, Marten R, Minocha K, Johnson C, Wong H, et al. Strategies to adapt and implement health system guidelines and recommendations: a scoping review. Health Res Policy Syst. 2022. 10.1186/s12961-022-00865-8. 35706039 PMC9202131

[6] Vélez CM, Velásquez-Salazar P, Vera-Giraldo CY, Velásquez-Correa JC, Santiago-Franco J, Lugo-Agudelo LH, Vélez-Marín V, Fahim C, Marten R, Yangchen S, Straus S. Developing a strategy for identifying recommendations prioritized for implementation in the Colombian health system. Health Res Policy Syst. 2026. 10.1186/s12961-026-01465-6.

[7] Lavis JN, Permanand G, Oxman AD, Lewin S, Fretheim A. SUPPORT tools for evidence-informed health policymaking (STP) 13: preparing and using policy briefs to support evidence-informed policymaking. Health Res Policy Syst. 2009;7:507–13. 10.1186/1478-4505-7-S1-S13 PMC3271824 20018103

[8] Vélez CM, Vélez-Marín VM, Lugo-Agudelo LH, Patiño-Lugo DF, Velásquez-Salazar P, Mesa-Franco LF, Vera-Giraldo CY, Velásquez-Correa JC, Fahim C, Marten R, Yangchen S, Straus S. Using evidence briefs to support implementation planning: early fibrinolysis and rehabilitation for the amputee patient in Colombia. Health Res Policy Syst. 2026. 10.1186/s12961-026-01466-5.

[9] Adjorlolo S, Ohene LA, Chandi MG, Aryeetey C, Ansah-Ofei AM, Aikins M, Aziato L. Challenges of home visit practice and perceived solutions for improvement in the Ghanaian context: perspectives of healthcare professional stakeholders. Health Res Policy Syst. 2026. 10.1186/s12961-026-01461-w.

[10] World Health Organization. WHO guideline: recommendations on digital interventions for health system strengthening: evidence and recommendations. Geneva: World Health Organization; 2019. p. 1–150. 31162915

[11] Adewuya AO, Abdulmalik J, Abimbola S, Oladipo E, Dahiru A, Shettima F, Fasawe A, Fahim C, Marten R, Yangchen S, Straus S. Adaptation and pilot implementation of the WHO guideline on digital interventions to strengthen mental health systems in Lagos State, Nigeria. Health Res Policy Syst. 2026. 10.1186/s12961-026-01463-8.

[12] Graham ID, Logan J, Harrison MB, Straus SE, Tetroe J, Caswell W, et al. Lost in knowledge translation: time for a map? J Contin Educ Health Prof. 2006;26(1):13–24. 16557505 10.1002/chp.47

[13] Mwamba C, Banda T, Mazaba M, Mukonka V, Malama K, Hamweemba E, Syulikwa M, Chilengi R, Sharma A, Fahim C, Marten R, Yangchen S, Straus S, RAISE Project. Raising the bar: translating global guidelines to achieve local policy on cholera elimination in Zambia—A qualitative case study. Health Res Policy Syst. 2026. 10.1186/s12961-026-01464-7.

[14] Fahim C, Pratt J, Purewal A, Marsot-Shiffman L, Quinn de Launay K, Baddeliyanage R, Davenport-Huyer L, Yangchen S, Gebregiorgis AH, Wiysonge CS, Tricco AC, Puchalski-Ritchie L, Pham B, Tuncalp O, Langlois EV, Marten R, Straus SE. Implementation and evaluation of capacity strengthening activities for the Research to Enhance the Adaptation and Implementation of Health Systems Guidelines (RAISE) in LMICs. Health Res Policy Syst. 2026. 10.1186/s12961-026-01462-9.

